# Significance of Participants’ Expectations in Managing the Placebo Effect in Antidepressant Research

**DOI:** 10.3389/fpsyt.2019.00713

**Published:** 2019-10-01

**Authors:** Marko Curkovic, Andro Kosec

**Affiliations:** ^1^Department for Diagnostics and Intensive Care, University Psychiatric Hospital Vrapce, Zagreb, Croatia; ^2^School of Medicine, University of Zagreb, Zagreb, Croatia; ^3^Department of Otorhinolaryngology and Head and Neck Surgery, University Hospital Center Sestre milosrdnice, Zagreb, Croatia

**Keywords:** depression, antidepressants, placebo, placebo effect, expectation, expectancy, research methodologies and methods

## Expectations as Major Determinants of the Placebo Effect

Unraveling complexities behind the response to treatment marked the placebo effect as a legitimate subject of scientific inquiry, even though it was conceived as a safeguard against the bias and uncertainty that accompany biomedical research (1–3). As the placebo effect comes under scrutiny regarding treatment specificity, it has been postulated that most of its effects are driven by participants’ expectations (4, 5). Such an argument has substantial empirical backing, and the placebo effect is now commonly referred to as an expectation-related effect. Attempts to distinguish it from other possible effects related to scientific methodology in general (regression to mean, the course of the disease, response bias, etc.) are numerous (1, 6). Participants’ expectations are defined as beliefs about the nature and the possibility of improvement as a consequence of receiving therapeutic intervention (4). In this article, the terms *expectations* and *expectancie*s are used interchangeably, but it is important to note that they are commonly defined differently. Expectations refer to the measurable beliefs that can be verbalized by individuals, while expectancy refers to psychologically driven predictions that can exist and act without an individual’s full awareness (4, 7).

## Expectation-Related Placebo Effects and Antidepressants

The expectation-related placebo effect is especially relevant in the field of depression and related therapeutic intervention, as there are difficulties in distinguishing between specific and nonspecific factors of therapeutic intervention. Empirical findings imply that the placebo effect in antidepressant studies may be expectation-related. The probability of receiving placebo intervention (unbalanced group randomization) has been repeatedly and firmly correlated with response rates to antidepressant therapy (8–10). This probability remains the most robust mediator and moderator of the placebo effect in antidepressant studies, and the only one that survived repeated analyses (8–11). The lower the probability of receiving placebo intervention, the greater the response to antidepressant therapy. Similarly, the placebo effect is less present when there is a lower probability of receiving antidepressant therapy. Interestingly, this relationship has a linear, gradual distribution; as we move from greater toward lower probabilities of receiving placebo intervention, the efficacy and acceptability of antidepressant therapy increase (10). Consequently, for the same antidepressant, there are significantly higher response rates in comparator head-to-head studies than in placebo-controlled studies. This linear relationship has recently been proven in an independent, experimental setting: as investigators manipulated participants’ perceived probability of receiving antidepressant therapy, placebo and antidepressant responses changed as already stated (12).

This relationship may be implicit proof that therapeutic responses in antidepressant studies are driven by participants’ expectations, more closely, their reverse expectations—the study participants’ belief that they are assigned to the placebo intervention arm, which is supposed to represent a neutral intervention (8, 10, 11, 13). Similar relationships may also be identified across a spectrum of conditions and treatments and have been attributed to a genuine placebo effect (14). Importantly, if the placebo intervention represents a valid epistemological tool (currently under debate), these probabilities should be considered when extricating the true efficacy of antidepressants in placebo-controlled studies (10).

There are several issues with this line of argumentation. First, it disregards the relational and dynamic nature of the placebo phenomena. Secondly, it overestimates the impact of expectations and (un)conscious processes associated with the placebo effect. Finally, it places too much emphasis on study participants and disregards other more or less obvious sources of bias and error (moderators and mediators of placebo and antidepressant response and genuine effects).

## The True Role of Expectation-Related Placebo Effect in Antidepressant Studies

The placebo intervention is composed of a context-specific set of “objectives and procedures” aimed at singling out one specific aspect of treatment (presumably the true intervention) while controlling for all others (1). The placebo could be conceptualized relative to many different interacting features—participant-related, intervention-related, condition in question, underlying theoretical assumptions, and the broader social and cultural context. The cumulative placebo responses could be viewed as an uninformative sum of “apples and oranges.” A recent study demonstrated significant differences in eliciting placebo response between studies using antidepressants belonging to the same pharmacological group (15).

Within placebo explanatory research, it has been suggested that mechanisms driving the placebo effect could be based on expectation-based concepts (1, 4–6). These can be active and predominantly conscious responses to more or less salient contextual and/or internal cues (5). As stated previously, the placebo-expectation paradigm has sufficient empirical neurobiological foundation and emphasizes the human mind as a “prediction machine.” When sufficiently broadly defined, expectations may represent a common final pathway of the placebo effect (4, 7). However, other placebo effect conceptualizations put more emphasis on the relational and broader contextual determinants, highlighting silent priors and underlying unconscious processes (1, 5, 6, 16). In alternative frameworks, the placebo effect either refers to dimensions other than expectations or is considered redundant in precisely describing phenomena driving the therapeutic response (6, 16–18). The relationship between patients and practitioners has been considered an essential part of the placebo effect, representing the process of interpersonal healing (18, 19). This is relevant in the field of psychotherapy where “common factors” such as therapeutic alliance, empathy, positive regard, and affirmation drive most of the therapeutic effects regardless of intervention type (20). Following a similar line of argumentation, the concept of the “care effect” has been proposed instead of the placebo effect (18). More recently, the placebo effect has been described as an outcome of persuasive communication, where the practitioner characteristics exert significant influence on the placebo effect (21). Additionally, emphasis on various contextual features of the treatment situation that have profound therapeutic effects brought out the concept of “contextual healing,” with the “meaning response” representing an underlying mechanism (16, 18, 22). Mindsets and different forms of social learning have also been singled out as crucial in understanding how broader social context is shaping the placebo effect (23).

Empirical findings in the field of open-label placebo administration (without a conscious intention to deceive) provide a good example of the flaws related to the expectation paradigm (7). The placebo intervention, administered in open-label fashion, may drive positive therapeutic effects in depressed individuals (24). Furthermore, it seems that placebo effect can occur even if a person lacks cognitive or epistemological resources required to form consistent belief and consequent expectation (25). It has been shown that participants’ expectations of intervention assignment change significantly while retaining their predictive relationship to measured outcomes (26). It seems that only expectations arising after initiating treatment have predictive potential, while those present prior to treatment are not informative (27). This is somewhat at odds with findings that single out inherent inflexibility and rigidity of negative expectations as a core feature of depression (28). These pathologic expectations are perpetuated and remain refractory to any form of adaptation (28). In other words, expectations may be deconstructed to trait-like (inter-individually) and state-like (intra-individually) variable components while retaining different precursors, moderators, mediators, predictive power, and manipulative potential (4, 14, 27). Finally, expectations are dynamic, are time and context dependent, and may involve higher-order and lower-order mechanisms that decode contextual information (7). They actively influence the one that is expecting, while trying to reconcile what is expected and what is experienced. The brain is more than a passive decoder, and perception seems to be an inferential process (5, 7).

One cannot truly praise the role that expectations play in the placebo effect without considering its “dark side”—the nocebo effect. As such, the nocebo effect includes adverse effects that are not attributable to characteristic features of treatment intervention (5, 28). Although underlying mechanisms of placebo and nocebo effects only partially overlap, it seems that the nocebo effect is mostly driven by negative expectation and learning (such as symptom misattribution, social transmission, etc.) (5, 28, 29). Nocebo-related adverse effects are prevalent in antidepressant studies (Mitsikostas et al. estimated 44.7%, while a more recent study by Dodd et al. quoted 63.7%) (30, 31). In antidepressant studies, patterns of nocebo-related adverse effects closely resemble adverse effects occurring in the active group (29–33). A meta-analysis by Rief et al. demonstrated that significantly greater tolerance of selective serotonin reuptake inhibitors (SSRIs) when compared to tricyclic antidepressants (TCAs) was identical in both groups, those receiving placebo and active treatment alike (34). These findings are indicative that awareness of adverse effects may influence patients’ expectations. A recent study on healthy participants demonstrated that adverse effects of antidepressants can be learned and reproduced. After participants initially received 50 mg of amitriptyline through a 4-day period, later administration of placebo also provoked amitriptyline-specific side effects (35). Cognitive bias in attention (sustained attention to negative information and cues), interpretation (tendency to interpret ambiguous information as negative), and memory processes (preferential recall of unduly negative memories) are considered as core feature of depression, playing a crucial role in the onset, maintenance, and recurrence of the illness (36). So, it is not surprising that depression has been singled out as one of the most important psychological factors contributing to the nocebo effect. However, when comparing depression to other brain diseases, such as motor neuron disease, Parkinson’s disease, and Alzheimer’s disease, all of the latter seem to have more prevalent nocebo adverse events than the ones found in depression (33). However, it remains a question whether nocebo phenomena are disease specific or treatment specific.

Study participants in antidepressant studies are a unique population, substantially different from the population antidepressants are aimed at. These individuals live in differing contexts; have differing motivations behind their participation; and have differing disease trajectories and previous treatment experiences, healing capacities, self-management strategies, and illness-related behaviors (13, 37–39). They undergo different informed consent, recruitment, randomization, and initiation procedures. Subsequently, they receive a different amount of intended or unintended attention and have differing capacities to understand the true nature of the study situation (5, 13, 37, 39–41). One should keep in mind the high prevalence of therapeutic misconception (a false belief held by participants that they are receiving the best possible care) in mental health research and different surrogate decision-making strategies (42, 43). Here the concept of placebo effect by proxy could be of some relevance, as it recognizes the potential impact generated by participants’ broader relational context.

**Figure 1:**
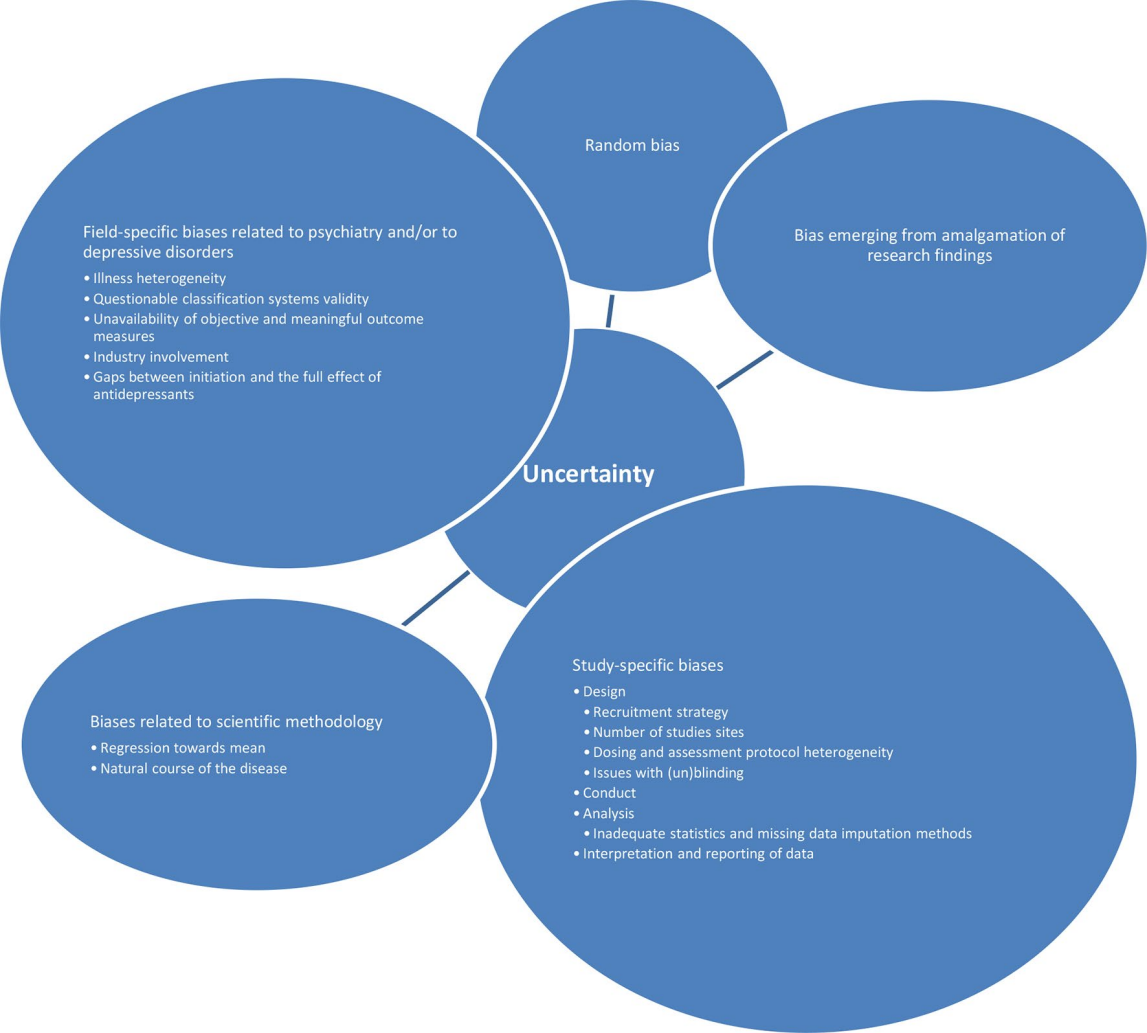
Sources of uncertainty that are mutually synergistic and non-additive.

Further on, antidepressant studies seem to be especially vulnerable to other sources of errors and biases: 1. random biases; 2. biases related to scientific methodology in general (such as regression toward mean, the natural course of the disease, etc.); 3. field-specific biases related to psychiatry and/or to depressive disorders; 4. study-specific biases (related to design, conduct, analysis, interpretation, and reporting of study data); and finally, 5. biases emerging from amalgamation of research findings. A number of possible sources of field-specific biases could differentially influence the placebo effect and/or antidepressants effects. Those include illness heterogeneity, classification systems’ validity, unavailability of objective and meaningful outcome measures, waxing and waning course of illness, industry involvement, and gaps between initiation and the full effect of antidepressants (1, 2, 11, 13, 37, 37, 38, 41, 44). Additionally, possible study-specific biases include different initiation periods and methods, recruitment strategies, number of study sites, study duration, dosing and assessment protocol heterogeneity, issues with (un)blinding, etc. (2, 11, 13, 37–39, 41, 44–46). Possible sources of biases within analysis (such as using inadequate statistical and missing data imputation methods), interpretation (such as one-sided interpretations primarily serving initial study purposes), and reporting (such as publication bias, or more specifically underreporting of studies with negative outcomes) of the studies’ data have been well documented (3, 11, 13, 37–39, 41, 44, 46, 47). An additional layer of complexity stems from findings that imply that all these sources of uncertainty seem to be synergistic and non-additive (48) (**Figure 1**). Any of the abovementioned sources of uncertainties has its own unique way of influencing the treatment response. Some of them may have comparable impact, and some may have different impact on drug and placebo response. Some may predominantly influence the placebo response, while others may have specific influence on the drug response. Some may narrow the gap between the placebo and the “true” response, and some could do the opposite. Importantly, it seems that the participants’ expectations about the future treatment effects do exert a systematic influence, above and beyond those sources of uncertainties. The researchers’ aim is to abstract and control all possibly important influences and to extrapolate the specific effects that are driving the total therapeutic response.

## Discussion and a Way Forward

Although unbalanced group randomization has been reported as the most reliable mediator of the placebo effect in antidepressant studies, attempts to eliminate sources of uncertainty reveal a daunting task in parsing out the exact role of the placebo and true antidepressant response (1–3, 5, 6, 10, 39, 47, 49, 50). These findings imply that the placebo effect is strongest in comparator antidepressant studies, where it cannot be reliably approximated. If the genuine placebo effect is driven mostly by participants’/patients’ expectations, strategies concerned with expectations’ modification are needed to maximize its positive effects. This approach has recently been questioned within the concept of “paradox expectations,” proposing that unfulfilled expectations could provide an adverse effect (7, 39, 51). More recent findings report discrepancies between current depression severity and in-treatment expectations that predict higher depressive symptom reduction (27). The more unrealistically optimistic expectations are, the greater the benefit they generate.

However, it seems highly unlikely that only one mechanism is responsible for a complex phenomenon behind the placebo effect. Exaggerating the importance of participants’ expectations could also steer us away from understanding the phenomena that may exert a systematic influence on possible sources of uncertainty. Mechanistic and reductionist approaches are of no use in furthering our understanding of these influences. A form of “self-fulfilling prophecy” arising from the investigators (trying to navigate themselves and others through the sea of competing interests) and their broader social context may be of greater relevance here. It has been already shown that the investigators’ expectations can have significant impact on study outcomes (7). Such a concept redirects responsibility toward agents that have capacities and conditions necessary to exert control over possible sources of uncertainty. There are different strategies on how to objectivize investigators’ related expectations and biases, but most of them include *post hoc* analytical tools with limited usefulness. The investigators may have discrete, unintentional expectations that are extremely difficult to objectivize. So, this shift of responsibility is of particular relevance in an ongoing debate concerning basic premises of scientific epistemology—rigor, reproducibility, transparency, and applicability. Scientific scrutiny needs to be simultaneously directed to all possible sources of uncertainty, participant and investigator, as well as context and trait- and state-like sources of uncertainty. Consequently, it seems that the only way to understand the genuine therapeutic effects in depression is to approximate all possible (non)specific factors that can influence outcomes of interest (2, 3, 39, 50). The nocebo effect also plays a significant role in the comprehensive evaluation not only of a treatment intervention’s safety but also of its efficacy and effectiveness. This is especially relevant for the interventions that are usually used over longer periods of time, with a significant proportion of the population being exposed to its effects (whether directly or indirectly). Therefore, equal attention should be placed on the nocebo effect during design, conduct, and analysis of the research as has been given to its more famous counterpart. One needs to remember that the placebo and nocebo effects only partially overlap and do not exert unanimously opposite influences. Finally, positive and negative expectations are fundamentally different things, in neurobiological, psychological, and social settings.

## Conclusion

In conclusion, as participants’ expectations play a significant role in the placebo effect, they should be evaluated systematically. Although there are still no firm recommendations in how to measure participants’ expectations, and the Hawthorne effect remains ubiquitous, expectations should be assessed before the initiation of intervention, several times during the study’s course, and ideally during the follow-up period as well. Such objectivized findings should then be weighed against other mediators and moderators of treatment. This may seem counterintuitive, but more recent systematic evaluation of expectations in depression treatment suggests limits that exist in the expectation-placebo paradigm. Finally, we know much about underlying (neuro)biological mechanisms of expectations, and participants’ expectations can indeed be manipulated and hopefully used in clinical practice, fulfilling the goal of translating placebo-related effects into clinical practice.

## Author Contributions

MC provided initial idea and construct of the manuscript. MC and AK co-authored and edited the manuscript.

## Conflict of Interest

MC has received lecture honoraria from Lundbeck, Sandoz, Janssen, and Alkaloid.

The remaining author declares that the research was conducted in the absence of any commercial or financial relationships that could be construed as a potential conflict of interest.
